# Foreign Summary

**Published:** 1839-06-08

**Authors:** 


### FOREIGN SUMMARY.

*A Lecture on Rheumatism.* By Robert Carswell, M. D.—Gentlemen: I propose to lay before you a summary of the cases of rheumatism at present under my care in the hospital. Some of these have been under treatment for a considerable time, while others have been but recently admitted. In two of the former, the acute general symptoms have disappeared, and the disease presents itself in the chronic form; and in one other of the same class there remain only the local sequelæ of the disease. Of the recent cases I shall notice one only, as a good example of acute rheumatism, in which the local and general symptoms are well marked, although not of great severity, and in which there is also the important complication of a simultaneous affection of the sero-fibrous structure of the heart.

Although the most of you are, no doubt, aware of the several forms under which rheumatism occurs, I shall briefly state the more important of them, as we have examples of each in the cases to which I shall allude.

And, in the first place, rheumatism is divided into *acute* and *chronic*. These two terms, however, express merely a difference in the degree and duration of the disease; the line of demarcation between the two not being always very distinct, inasmuch as the fever and local inflammation are present in both, and vary much in relation to each other in degree, also, in both. With regard to the chronic form of the disease, it may either succeed to the acute, or it may have been so from the commencement. And, again, with regard to the local symptoms which accompany either acute or chronic rheumatism, these may persist after the general symptoms have disappeared in both; that is to say, the local inflammation, particularly of the joints, which occurs in both the acute and chronic forms, may continue for an indefinite period after the febrile state has been removed by proper treatment. And it is to this stage or form of the disease that the appellation of *hot* and *cold* has been applied, which bear the same import as the terms *sthenic* and *asthenic*. The *hot*, or *sthenic* form has also been distinguished by the term *active*; and the cold, or asthenic, by that of arthrodynia.

Lastly, what are called the sequelæ of rheumatism, *viz.*, enlargement, thickening, induration, and other morbid states of the joints, may remain after the local inflammation which had produced them has disappeared, and without being accompanied by any disturbance of the general health. But I need hardly observe that these local morbid conditions can no longer be associated with rheumatism, either as regards their nature or treatment, no more than similar changes occurring in any other part of the body as the consequence of ordinary inflammation, and from which, so far as we know, they differ only in their having, at their commencement, been associated with rheumatic fever. I may also notice here, as a very common effect of rheumatism of the joints, defective muscular power, which is sometimes so great as to prevent entirely the patient from using the affected extremities, even after all pain and swelling have disappeared. This was the case particularly in the hands of one of our patients, Maria List, who could not grasp or support with her fingers and hands,—

although in no way deformed,—the crutches she was recommended to use to assist her in walking.

From these general remarks, the several forms of rheumatism may be enumerated as follow:— 1st. Acute rheumatism; 2d. Chronic rheumatism, sub-divided into chronic rheumatism, consisting first, in fever and local inflammation; secondly, in local inflammation without fever: and, 3rd, into the sequelæ of rheumatism, or various morbid states of the joints.

Before alluding to the individual cases, and the treatment of them, I may also notice an affection which, whether existing in an acute or chronic form, has been considered by many physicians of a rheumatic nature, viz., *sciatica*, of which we have now two cases under treatment. That this painful affection is frequently of a rheumatic nature, there hardly can exist a doubt, inasmuch as it is met with accompanying the usual forms of acute or chronic rheumatism; sometimes preceding, at others following an attack of this disease. In other cases, again, it can in no way be associated with rheumatism, but, like rheumatism, is an acute inflammatory disease, requiring the same active antiphlogistic treatment, both local and general, at its commencement, for its cure; and, indeed, morbid anatomy has demonstrated the inflammatory nature of this disease in certain cases, the sheath of the nerve having been found in a state of high inflammatory congestion, and the nerve itself softened or converted into a mere pulp.

The first case, then, which I shall notice, is one of acute rheumatism, occurring in a female, named Mary Bye, in the private ward, twenty-four years of age, stout, and of a sanguineous temperament. The disease made its attack under the ordinary circumstances, or under the influence of the ordinary exciting causes, viz., exposure to wet and cold, as a servant of all-work. It is stated that a fortnight since she first felt some pain in the left foot, which soon after became red and swollen; the pain soon attacked the knee and hips, and subsequently the hand. She has had leeches and fomentations, and taken purgatives.

*On her admission.*—The right hand is principally affected; the metacarpal joints of the first and second phalanges are highly inflamed and excessively painful, and she cannot open or shut the hand; she describes the pain as gnawing, sometimes darting or shooting. The pain is evidently very much relieved by cold, though very slightly increased by heat. The left ankle and knee are also similarly affected, but in a less degree; considerable pain is felt in them on the slightest motion or pressure. Both knees are swollen from œdema; skin hot and perspiring; face expressive of pain; countenance flushed, and eyes injected; great thirst; furred tongue; no appetite; bowels regular; urine high-coloured and scanty; menstruation performed regularly; pulse 98, full and firm.

*Heart.*—With the first sound is heard a bellows-sound, louder than that with the second; they are heard most distinctly at the base; rhythm and impulse natural.

Feb. 1. Bleeding to twelve ounces; acetous extract of colchicum, one grain, every night and morning. Low diet.

2. Blood buffed; pain very little less in any part.—Bleeding to 12 ounces.

*Calomel*, 3 grains;
*Comp. ipecac. powder*, 5 grains; every night and morning.

5. Blood both buffed and cupped; hand very much better; the elbow and shoulder are now the seats of severe pain. The patient obtains no sleep at night from the pain, which is then much worse than during the day.—Increase the extent of colchicum by half a grain, and the comp. ipec. powder to 10 grains.

6. Passed a sleepless night; very little improvement; gums slightly affected; bellows-sound heard much less, and with the second sound is hardly perceptible.

The most interesting circumstance to be noticed in this case is the occurrence of *endocarditis*, as indicated by the bellows-sound. This anormal sound accompanies both sounds of the heart, and is heard most distinctly at the base of that organ. That it indicates the presence of inflammation of the lining membrane of the valves, and most probably chiefly of the aortic valves, there cannot, I believe, be any doubt. It was heard at the time the patient was admitted, and may have existed for some time before. Although the case is, as I have already said, a good example of acute rheumatism, the symptoms are far from being so severe as they are frequently met with in this disease. Its being complicated with endocarditis is, therefore, the more interesting,—a circumstance, indeed, of very common occurrence, even in the mildest forms of acute rheumatism. Such a complication ought always to be looked for, and the energy and efficacy of the means of treatment regulated and tested by the stethoscopic signs which this, the most dangerous in its future consequences of all the forms of rheumatic inflammation, affords. It is gratifying to observe the favourable change which has already taken place in this case; although the general symptoms have undergone but little amelioration, the intensity of the bellows-sound has considerably diminished; indeed, that which accompanied the first sound is hardly perceptible; and this favourable change has been effected in the space of five days, at the end of which time, also, the constitutional influence of the mercury was manifested by the state of the gums. The result of the case, which must interest you from this circumstance alone, will be announced to you on a future occasion.

Of the two chronic cases to which I have alluded, both of them at the time of admission presented the characters of chronic rheumatism, and that form of the disease termed the asthenic, or cold rheumatism, the joints being the parts affected, the pain being rather relieved than otherwise by heat, and there being little or no febrile excitement.

The first of these patients, Esther Barnes, æt. 26, was admitted the 11th of December. She is of a sanguineous temperament, of regular habits; has lived in a damp situation, and been accustomed to hard work as a servant. She had an attack of acute rheumatism five years ago, in the left knee, which lasted six weeks. Fourteen months ago she had another attack in both knees, which became contracted, and have remained much in the same state ever since. The symptoms at her admission were the following:—Temperature of surface natural; suffers much pain in both knees; great effusion in the joints; pain not increased by heat; is worse when she attempts to move; sleeps badly; respiration and sound of heart natural; tongue clean; bowels regular; urine natural; catamenia regular. On the same day it is afterwards stated that the "rheumatism is relieved by heat."

This, then, is a good example of the cold or asthenic form of chronic rheumatism, marked as well by the duration of the disease as by the relief afforded by the application of heat to the affected parts, and the nearly natural state of all the important functions of the body.

Notwithstanding the length of time which the disease had lasted, and the great enlargement of both knees, rendered it more than likely to be one that would yield but slowly to any mode of treatment, the case has, however, gradually improved; the pain and swelling of the knees have gradually subsided. Indeed there is no pain, unless when the patient moves the limbs, as in walking, which she can now do with greater ease.

The treatment adopted consisted in the internal use of the iodide of potassium, and the repeated application of blisters, sinapisms, and stimulating liniments. She began with ʒss. of the solution of the iodide of potassium, three times a day, which was increased, in the course of three or four weeks, to ℈iv., when it was laid aside. The treatment now consists of local applications alone. The patient has been nearly two months in the hospital.

The next case is that of Anne King, æt. 45, who, about two years ago, after getting wet, had rheumatism in the hands and knees, and since which time she has never been altogether free from it; on the contrary, it has been gradually aggravated during the last eight months. On her admission she presented the following symptoms:—

Skin hot and dry; feet and hands always cold; complains of a gnawing pain in hands and knees, which is less when hot than cold; great pain is produced by pressure; hands are slightly swollen; fingers flexed and not extensible; effusion into the knee-joints, more so in the left; both legs flexed, and incapable of extension; chilblains on the toes of both feet; bowels regular; catamenia ceased a year ago; tongue very white, but moist; great weakness in loins and hips; urine plentiful and high-coloured; pulse 80, and regular.

The treatment in this case was similar to that adopted in the preceding. The iodide of potassium was employed, beginning with ʒss. of the solution three times a day, and gradually increasing the dose. At the end of a fortnight she was taking ʒi. of the solution, and was much improved in all respects.

On the 10th of January the pains became worse, which was attributed to change of the weather, and continued so for a week, when the iodide of potassium was laid aside, and the ammoniated tincture of guaiacum and decoction of bark substituted. This plan of treatment has been continued up to our last visit, together with the local application of stimulating liniments, sinapisms, and occasionally poultices, but with very little improvement. As the patient complains much of weakness and loss of appetite, she has been ordered one grain of the sulphate of cinchonine and one ounce of the infusion of absinthium, three times a day, and to continue unremittingly the external applications.

The third case to which I alluded is that of Maria List, æt. 23, who was admitted more than three months ago, and the history of whose case is rather ambiguous. She complained chiefly of loss of the use of the extremities, from the feet to the knees, and from the hands to the elbows; pain in the lumber region; inability to stand or take a hold of anything. She was bled twice to twelve ounces, within the first four days, and took the colchicum wine during the first week, with relief of the pain. On the 30th, that is two weeks after her admission, she was put upon a course of the iodide of potassium, soon after which the pain disappeared, except in the joints, when extension of them was attempted. Blisters, cataplasms, and stimulating liniments have been applied to the knees; extension of these has been gradually effected; the fingers and hands are regaining their power; and the patient is now able to walk about with the assistance of crutches.

It was to the loss of power and motion in this case, to which I alluded, as a good example of these morbid conditions as the consequences of chronic rheumatism.

The cases which I have now to bring under your notice are two of rheumatic inflammation of the sciatic nerve, or sciatica. They are both very marked cases of the affection, and although the patients have been attacked more than once, they never have had rheumatism of the joints.

The first case is that of James Dalgleish, admitted the 15th of this month. He is a groom, of sanguineous temperament, florid complexion; married; of pretty regular habits; living in London; has always enjoyed very good health. His father died of calculus vesicæ; his mother is still living and healthy.

*History.*—In August last he was affected with rheumatism, occupying the right leg, and extending from the hip to the ankle. It left him in about four days under the use of spirit. terebinth. as a liniment. He can assign no cause for the attack. He then continued free from it until the 5th December last, when, after having been out all day in a chaise, when the weather was very cold, he was again attacked with rheumatism in the same limb, and to the same extent, but more severe than before. It now prevented him from walking or sitting, but he was quite easy when lying down. The pain was relieved by warmth; he has derived no benefit from the treatment he has been recommended.

*Present symptoms.*—He now complains of a dull aching pain as soon as he attempts to stand or sit, which gradually becomes more and more severe, and compels him to lie down, which gives him immediate relief. The pain leaves him entirely when he is in bed, the affected limb only feeling somewhat numb. Warmth relieves the pain, cold increases it. The skin is hot and dry; pulse 80, and full; tongue whitish and slightly furred. A little cough and expectoration for a few minutes at first getting out of bed in the morning; bowels regular; some difficulty experienced in making water when the pain is severe; urine scanty and dark-coloured, and deposits a thick sediment. The joints of the affected limb are not swollen or red.

Jan. 15. *Calomel*, 5 grains;
*Compound extract of colocynth*, 6 grains. Make two pills to be taken at bed-time.
*A senna draught* in the morning.

Venesection to 12 ounces; cupping to 12 ounces over the hip.
*Colchicum wine*, 20 minims;
*Carbonate of magnesia*, 1 scruple;
*Camphor mixture*, 1 ounce. To be taken thrice a day.

19. Blood buffed and cupped; the serum of that taken from the arm was very milky. A great deal of relief has been derived from the cupping; there being now no pain felt except in moving, and is then felt chiefly in the ham-string and muscles.—A blister over the sacrum.

22. Pains rather worse, increased in the morning; bowels regular.—Repeat blister; to be kept open with savine ointment.
*Comp. ext. of colocynth*, 10 grains;
*Calomel*, 2 grains. To be taken at bed-time, and repeated if necessary.

26. Pain much less, and only felt when walking, or after standing for some time.

29. Improving; blister allowed to heal on the 25th; is not purged by the colchicum, but the bowels are kept open by the calomel and colocynth pill taken every night.—Increase the colchicum wine to 40 minims.

Jan. 2. For the last three days there has been more pain. There is also some cough, in consequence of the patient having taken cold. The acetous extract was now substituted for the wine of colchicum, a grain of the former being ordered to be taken three times a day. In consequence of the cough, and in order to procure sleep, a pill, composed of five grains of henbane, and ten grains of Dover's powder, was also ordered to be taken at night. Such is the state of this patient's case, and the treatment up to the present time.

The second case is that of John Withers, æt. 40, admitted the 21st of the same month. The patient has had two similar attacks, the first about twenty-two, the second five years ago. The first was the more severe of the two, and continued for two months. The history and symptoms of the present attack are as follow:—Three weeks ago, after having been exposed to cold and wet in the pursuit of his employment, (house-painting,) he felt the rheumatism coming on in his left hip, although slightly; and on the 14th of this month he got wet through, and allowed the clothes to dry on him. The next day he could not move for the pain and stiffness of the joint. He got gradually worse, and was admitted the 21st January.

*Present symptoms.*—He now complains of most intense pain, extending from the sacrum down to the foot, the hip, knee, and ankle, being most affected. None of these parts are swollen or red, but feel very cold, and the patient has frequent shiverings. There is a continual aching pain in the joints which is relieved by cold and increased by heat. The skin is cool and natural; no perspiration: tongue whitish and moist; appetite very good; bowels rather costive; urine dark, rather scanty, and deposits a sediment.—Cupping over right hip to 10 ounces.
*Acetate af colchicum*, 1 grain, night and morning. Low diet.

22. Some relief in the hip from the cupping, but the pain still continues in the knee and ankle.
*Dover's powder*, 10 grains;
*Calomel*, 2 grains; every night. Continue acetate.

23. Somewhat better; slept for five hours last night, which he had not done for a week before.

26. Hip better, but the pain still continues in the ham and ankle. Ten leeches to the left ham. The Dover's powder to be taken only every other night. Middle diet.

31. The pain has left the hip and ham, but is very severe in the ankle, which is red and swollen.—Eight leeches to the right ankle.

Feb. 2. The pain much the same in the ankle, but has returned to the hip.—Ten leeches to hip; six leeches to right ankle.
*Dover's powder*, 6 grains;
*Calomel*, 2 grains; night and morning.

There is nothing requiring particular observation in the history of these two cases of sciatica. The disease is equally well marked in both, but of greater severity in Withers, and occurred in both patients under the influence of the ordinary exciting causes, viz., exposure to cold, or wet and cold. The mode of attack, and the symptoms have been very similar in both. There is, however, mentioned a singular difference in regard to the pain, which, in the first patient, is increased by cold, and relieved by heat; whereas, in the second, it is quite the reverse, the pain being relieved by cold, and increased by heat; and this is the more remarkable as the same nerve is affected, and the character of the pain is the same in both cases. It is a difference which I shall not attempt to explain, and could not, certainly, be taken as an indication of treatment when contrasted with the other symptoms present.

In two cases, so very similar in almost all respects, I have been desirous of testing the curative effects of the wine of the seeds, and of the acetous extract of colchicum.

In the case of Dalgleish considerable improvement followed the bleeding, the pain on the four following days being felt only while walking or moving the extremity. During the following week, however, it again became worse; afterwards less, and for the last three or four days, remained stationary, although the colchicum wine had been increased to 40 minims three times a day. It was in consequence of this latter circumstance that the acetous extract was substituted for the wine of colchicum, in the hope of effecting a more speedy cure of the disease. How far this will be accomplished by this alteration, you will have an opportunity of observing.

Less improvement has been obtained by the treatment in Withers's case, although it has been the same as in the former case, with this difference only that the acetous extract instead of the wine of colchicum, was employed. The pain is much less severe, but it shifts, when relieved by leeching, from one part of the affected extremity to another, and will, probably, prove more obstinate than in the preceding case.

[The first of these patients was discharged cured on the 12th of February. The pain disappeared the day after the use of the acetous extract of colchicum, and did not return; the henbane and Dover's powder were, however, continued till the 9th, along with the colchicum, and, probably, assisted in expediting the cure. The other patient did not leave the hospital till the 23d, and then at his own request, and still suffered from considerable pain in the hip. Various means were employed, subsequently to the use of the acetous extract of colchicum, and when it no longer appeared to afford relief. The chief of these were the mistura guaiaci, the tinct. guaiac. ammoniat., the hip-bath, and acupuncture; each of which was followed for a short time by some diminution of the pain, (which was principally seated in the hip,) which, however, soon returned, and at last became stationary. At the end of a week after leaving the hospital this patient was readmitted, and was again under treatment for upwards of three weeks, the disease being, in all respects, as severe as on the previous occasion. In addition to the means formerly employed, he took the spirit. terebinth. for some time, and afterwards the ferri carbon. in large doses, with no great apparent advantage from either. Ten grains of Dover's powder, with the addition of half a grain of opium, were taken night and morning, and to this part of the treatment the patient ascribed the chief benefit he derived. He was this time "discharged cured."]

I take this opportunity of showing you in this patient, (the patient was brought into the theatre,) who was admitted for disease of the heart and bronchitis, a very good example of that form of cutaneous affection called by Willan, pityriasis versicolor, and by other dermatologists, *chloasma, maculæ, hepaticæ*, &c.; you perceive, principally on the chest and upper part of the abdomen, a number of spots and patches of very various dimensions, and of a yellowish-brown colour. A few of them are not larger than the surface of a split pea; the greater number of them vary from one to two inches in breadth, and several are considerably larger. The spots are of a round or oval shape; the patches are more or less irregular in their shape, and are obviously the result of the union of smaller ones, segments of which are seen forming the circumference of the former. The discoloured surfaces are smooth, except here and there, where there is a very slight furfuraceous desquamation; and when the hand is passed over them, it is only the larger ones which are felt to project slightly above the surface of the surrounding skin. They are the seat of a certain degree of pruritus, which is always increased by external warmth, by whatever excites the general circulation, as active exertion of all kinds, stimulating food, and spirituous liquors. Under these circumstances the sensation of itchiness is sometimes very annoying, and compels the patient to have recourse frequently to rubbing or scratching the affected parts to obtain relief. Generally, however, the itchiness in this affection is not so troublesome, and the chief annoyance is the unseemly appearance of the skin, more especially when the discolouration affects parts which cannot always be concealed by the dress.

The duration of this cutaneous affection is extremely variable. It has appeared to me that it is found to exist for a much longer period in males than in females. In the former I have known it to have existed for many years; and in the present case it has existed several years; whereas, in the latter, it not unfrequently disappears after a few weeks, or even a few days duration. It must, however, be observed, that in such cases the cutaneous discolouration has occurred in females at the menstrual periods, on suppression of the menses, or during pregnancy; and, hence, this form of the affection has been by some pathologists denominated *chloasma amenorrhicus*, and *chloasma gravidarum*. Its real or supposed occurrence in diseases of the liver (of which I have not met with an example,) has obtained for these patches the name of maculæ hepaticæ. It may no doubt occur along with disease of this or other organs; but it is frequently met with in persons apparently in good health, and who apply to the physician only in consequence of the itchiness and discolouration of the skin. The real nature and cause of the discolouration are not known. It is wrong, however, to assign to it an inflammatory origin, and to class it with the squamous diseases of the skin, as has been done by Willan and Bateman. By these authors it has, as I have already said, been called *pityriasis versicolor*. But pityriasis is, essentially, a chronic inflammatory affection of the skin; accompanied for the most part by some degree of inflammatory redness, by increase of temperature of the affected parts, and constantly by desquamation, sometimes carried to a great extent. The discolouration of the affection of which I am now speaking, is not that which accompanies inflammation of the skin, and, therefore, ought not to be classed among the squamæ.

Its natural place is obviously among the *dischroa*, and whatever may be the causes to which it owes its origin, it appears to consist in an increase, or at least in a modification, of the colouring matter of the cutaneous tissue. This cutaneous affection, or chloasma, is, in general, easily distinguished from other similar affections. The obvious cause of *ephelis*, viz., exposure to the sun, and the appearance of the spots only on the parts thus exposed, are at once sufficient to establish a marked distinction between the two affections; and the distinction is made with the same facility in *lentigo*, from the freckles, as they are called, coinciding with the always reddish colour of the hair. *Nævi*, of a brownish colour, and which are not raised above the surface of the skin, bear some resemblance to circumscribed spots of chloasma, but are readily distinguished from it by the fact of their being congenital, and the absence of itchiness and desquamation. A much greater resemblance exists between chloasma and the *copper-coloured syphilitic patches*. From these latter, however it may also, in most cases, be distinguished by the previous history, by a difference of colour, and, *chiefly*, by the absence of pruritus.

The treatment of chloasma may be stated in a few words. The most effectual remedy is the sulphuret of potash, in the form of baths, or of vapour, and the sulphuric fume bath. I have seen the disease disappear entirely by one or other of these means, in a few weeks, that had resisted all others for years. Any complication must, of course, be treated apart, and those forms of chloasma which accompany disordered menstruation, or pregnancy, generally disappear on the removal of these states.—*Lancet.*

---

*Concours for the Chair of Organic Chemistry at Paris.*—The following brief notice of the *tests* to which the rival candidates *were submitted*, at the late *concours* for the chair of organic chemistry and pharmacology will probably be interesting to the American reader. The election by *concours* may, we will allow, be attended with some disadvantages, as it has been often remarked that professional and scientific men are generally the least impartial judges of professional and scientific merit; but that there are corresponding, and, we may add, overbalancing benefits from its adoption, cannot well be disputed.

The *first* examination was altogether occupied with the preparing of a written thesis upon the organic alkalis.

At the *second* examination, the candidates had to discourse orally, after twenty-four hours' preparation, upon a given subject—M. Baudrimont upon the chemical and pharmaceutical relations of alcohol; M. Bouchardat upon those of the essential oils; M. Bussy upon those of adipose and fatty matters; and M. Dumas upon those of sugar.

At the *third* examination—which was also an oral one, after two hours' preparation, upon a subject determined by lot—MM. Baudrimont and Bouchardat had to treat of the properties of albumen and gelatine, and MM. Bussy and Dumas upon those of milk.

At the *fourth* and last examination, the candidates were required to defend their respective printed theses—M. Bussy on the urine and its changes in diseases; M. Dumas upon the influence of heat on organic bodies, and its employment in pharmaceutical preparations; M. Bouchardat on the blood; and M. Baudrimont on the present state of organic chemistry.

Each candidate gave convincing proof of great knowledge and acquirements; but as the superiority of M. Dumas was acknowledged by all, he was unanimously elected to the professional chair.—*Med. Chir. Review.*

